# Optimal Preclinical Conditions for Using Adult Human Multipotent Neural Cells in the Treatment of Spinal Cord Injury

**DOI:** 10.3390/ijms22052579

**Published:** 2021-03-04

**Authors:** Jeong-Seob Won, Je Young Yeon, Hee-Jang Pyeon, Yu-Jeong Noh, Ji-Yoon Hwang, Chung Kwon Kim, Hyun Nam, Kyung-Hoon Lee, Sun-Ho Lee, Kyeung Min Joo

**Affiliations:** 1 Department of Health Sciences and Technology, SAIHST, Sungkyunkwan University, Seoul 06351, Korea; wjdtjq1124@gmail.com; 2 Single Cell Network Research Center, Sungkyunkwan University School of Medicine, Suwon 16419, Korea; kimck0405@gmail.com (C.K.K.); snutaeng@gmail.com (H.N.); leekh@skku.edu (K.-H.L.); 3 Stem Cell and Regenerative Medicine Center, Research Institute for Future Medicine, Samsung Medical Center, Seoul 06351, Korea; yeonjay.youn@samsung.com; 4 Department of Neurosurgery, Samsung Medical Center, Sungkyunkwan University School of Medicine, Seoul 06351, Korea; 5 Department of Anatomy & Cell Biology, Sungkyunkwan University School of Medicine, Suwon 16419, Korea; tadah881217@gmail.com (H.-J.P.); nyj1350@gmail.com (Y.-J.N.); sandyky1020@gmail.com (J.-Y.H.); 6 Medical Innovation Technology Inc. (MEDINNO Inc.), Ace High-End Tower Classic 26, Seoul 08517, Korea; 7 Biomedical Institute for Convergence at SKKU (BICS), Sungkyunkwan University (SKKU), Suwon 16419, Korea

**Keywords:** spinal cord injury, multipotent neural cell, neural stem cell, dose escalation, lateral ventricle

## Abstract

Stem cell-based therapeutics are amongst the most promising next-generation therapeutic approaches for the treatment of spinal cord injury (SCI), as they may promote the repair or regeneration of damaged spinal cord tissues. However, preclinical optimization should be performed before clinical application to guarantee safety and therapeutic effect. Here, we investigated the optimal injection route and dose for adult human multipotent neural cells (ahMNCs) from patients with hemorrhagic stroke using an SCI animal model. ahMNCs demonstrate several characteristics associated with neural stem cells (NSCs), including the expression of NSC-specific markers, self-renewal, and multi neural cell lineage differentiation potential. When ahMNCs were transplanted into the lateral ventricle of the SCI animal model, they specifically migrated within 24 h of injection to the damaged spinal cord, where they survived for at least 5 weeks after injection. Although ahMNC transplantation promoted significant locomotor recovery, the injection dose was shown to influence treatment outcomes, with a 1 × 10^6^ (medium) dose of ahMNCs producing significantly better functional recovery than a 3 × 10^5^ (low) dose. There was no significant gain in effect with the 3 × 10^6^ ahMNCs dose. Histological analysis suggested that ahMNCs exert their effects by modulating glial scar formation, neuroprotection, and/or angiogenesis. These data indicate that ahMNCs from patients with hemorrhagic stroke could be used to develop stem cell therapies for SCI and that the indirect injection route could be clinically relevant. Moreover, the optimal transplantation dose of ahMNCs defined in this preclinical study might be helpful in calculating its optimal injection dose for patients with SCI in the future.

## 1. Introduction

Traumatic spinal cord injury (SCI) results in the loss of both cells and connecting axons at the injury site, which interrupts the flow of information to and from the brain. Since transected axons cannot regrow or replenish these lost neurons, there is no naturally occurring pathway to repair the mature human central nervous system (CNS) [1]. This means that our ability to supplement the regenerative potential of the injured spinal cord is critical to recovery after SCI and that any technologies that may support these interventions have been at the center of the clinical research environment for the past 30 years. Advances in this field have propelled stem cell-based therapies to the forefront of innovative treatments for SCI.

Transplantation of neural stem cells (NSCs) has been shown to promote repair or regeneration of damaged CNS. Implanted NSCs replace lost neurons [2,3], create a favorable microenvironment supporting the regrowth of host axons [4,5], produce growth factors that protect damaged neural cells [6,7], and reform glial cells to support surviving neuron function [8]. We previously established an experimental protocol for the isolation and expansion of adult human multipotent neural cells (ahMNCs) from surgical brain samples [9,10]. These ahMNCs retain their self-renewal and differentiation capabilities allowing them to produce new neural cells, such as neurons and astrocytes, which are essential features of NSCs. Preclinical evaluation has shown that the transplantation of these ahMNCs into SCI sites demonstrates various therapeutic effects [11].

In a previous study [11], ahMNCs were cultured using surgical tissues isolated from patients with temporal lobe epilepsy and then directly transplanted into the SCI site. However, this preclinical protocol is not easily adapted to clinical settings for several reasons including 1) direct injection of ahMNCs into the injury site could cause unintended secondary injury [12], and 2) the characteristics of ahMNCs isolated from epilepsy sufferers could be different from those of ahMNCs isolated from brain tissues without abnormal electrical activity, and that these changes may influence their therapeutic effect in SCI. In addition, clinical trial requires that users establish a clinical guideline for ahMNCs dosage, which must be determined in a preclinical study.

This study was designed to address these issues and evaluate the use of cultured ahMNCs from patients with hemorrhagic stroke, and design and evaluate an indirect delivery route for ahMNCs to treat SCI animals. We determined that an intracerebroventricular (ICV) transplantation protocol using stem cells produced the best in vivo migration of injected cells into the disease sites of SCI via the cerebrospinal fluid (CSF) [13,14,15]. ahMNCs from hemorrhagic stroke were comparable to those from the temporal lobe epilepsy study demonstrating similar molecular and functional characteristics. When various doses, ranging from 300,000 (low) to 3,000,000 (high) cells/mL, of these cells were transplanted into the lateral ventricle (LV) of SCI animals, the cells exerted a significant effect on the locomotive functions and tissue recovery of these animals. Unexpectedly, the middle dose of ahMNCs (1,000,000) showed the most favorable outcome, which suggests that the optimal dose of stem cells for SCI treatment should be determined in a preclinical environment.

## 2. Results

### 2.1. Characteristics of ahMNCs from Hemorrhagic Stroke

**Figure 1:**
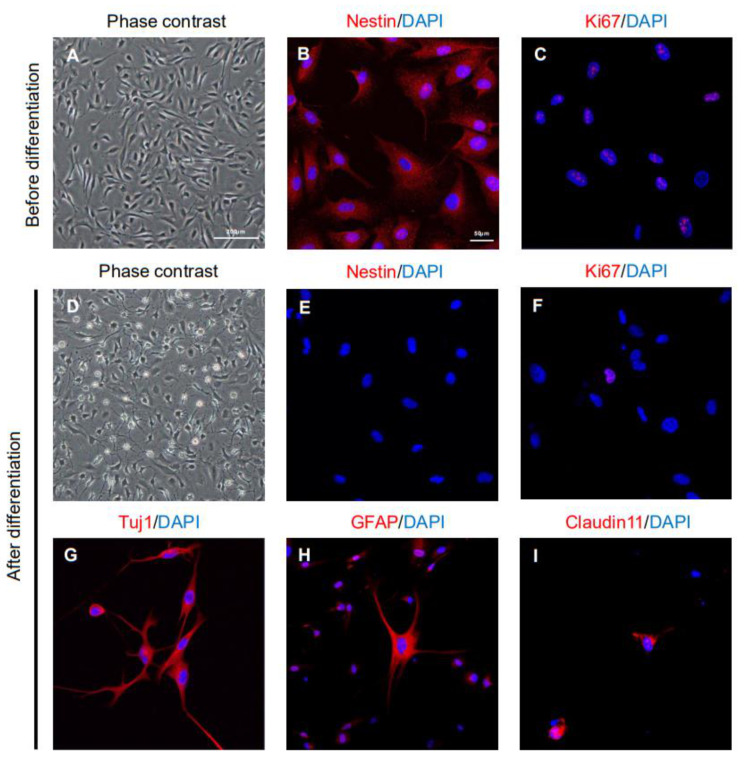
Characteristics of ahMNCs isolated from patients with hemorrhagic stroke. (**A**) ahMNCs were shown to be homogeneously bipolar and in the proliferation condition (before differentiation) at in vitro passage 6 (P6). (**B**,**C**) ahMNCs at P6 expressed the NSC marker, Nestin (**B**) and proliferation marker, Ki67 (**C**). (**D**) When P6 ahMNCs were subcultured under differentiation conditions (after differentiation), they demonstrated a range of morphological changes associated with differentiated neural cells. (**E**,**F**) The expression of Nestin (**E**) and Ki67 (**F**) decreased after differentiation. (**G–I**) Expression of differentiated neural cell markers such as Tuj1 (**G**, for neuron), GFAP (**H**, for astrocyte), and Claudin11 (**I**, for oligodendrocyte) was observed after differentiation. The scale bars = 200 and 50 µm in the phase-contrast and immunofluorescence images, respectively.

ahMNCs were isolated from the surgically excised brain tissues of patients with hemorrhagic stroke. These ahMNCs were primarily cultured under adherent culture conditions as previously described [10] and demonstrated the expected bipolar morphologies (Figure 1A). Nestin, a canonical NSC marker, was shown to be expressed on these cells (Figure 1B) and immunoreactivity to Ki67 confirmed their active proliferation (Figure 1C). Nestin and Ki67 expression were observed in almost every ahMNC before differentiation (Figure 1B,C), while their expression all but disappeared in ahMNCs treated with IBMX for differentiation (Figure 1E,F). These differentiated ahMNCs were shown to adopt the various morphologies associated with several types of differentiated neural cells (Figure 1D) and the expression of lineage-specific markers on these differentiated neural cells confirmed their identity (Tuj1 for neurons, Figure 1G; GFAP for astrocytes, Figure 1H; Claudin11 for oligodendrocytes, Figure 1I). These results indicate that ahMNCs retain many of the characteristics of NSCs, including self-renewal and differentiation into several neural cell lineages. These results were also similar to those described for the ahMNCs isolated from temporal lobe epilepsy [10].

### 2.2. Therapeutic Effects of ahMNCs on Spinal Cord Injury

**Figure 2:**
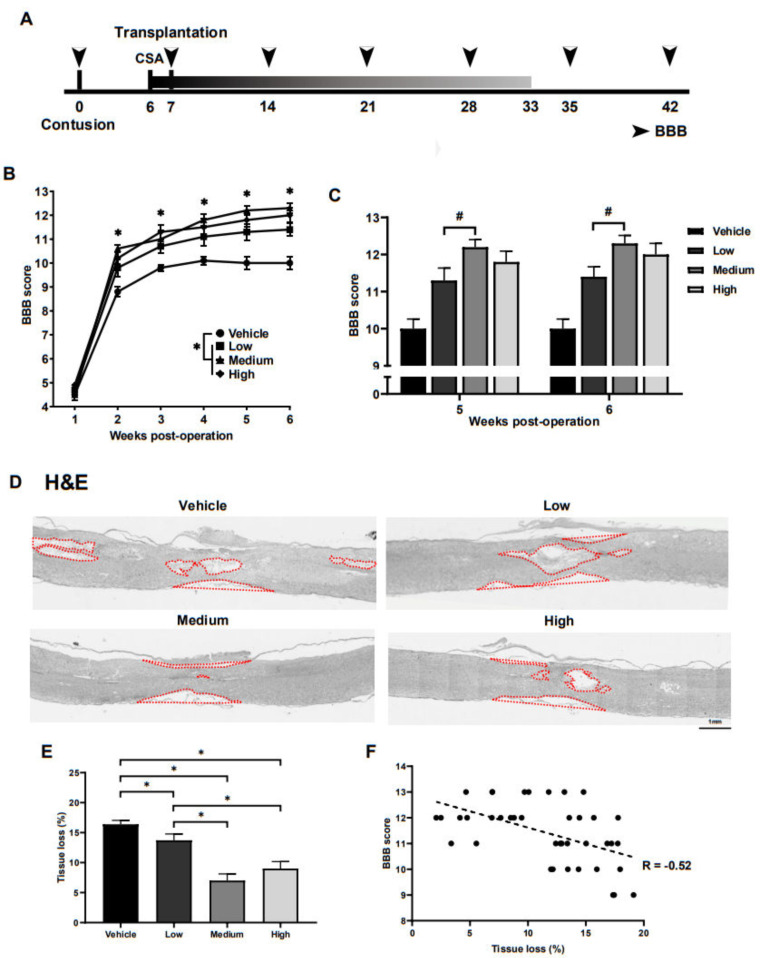
Preclinical therapeutic effects of ahMNCs in SCI. (**A**) Schematic describing the experimental timeline. (**B**) BBB scores were monitored once a week until 6 weeks post SCI. (**C**) The BBB scores at 5 and 6 weeks after SCI were compared. (**D**) Tissue loss was evaluated using histological methods at 6 weeks post SCI. Red lines delineate the contours of the lost tissue including both cavities and shrunken tissue. Scale bar = 1 mm. (**E**) Relative areas of tissue loss were quantified and then compared. (**F**) Correlation analysis revealed a significant negative relationship between the degree of tissue loss and the BBB score. Height = Average, Error bar = Standard Error. *, # *P* < 0.05.

To test the therapeutic effects of ahMNCs from hemorrhagic stroke on SCI, SCI was induced in the rat spinal cords, T9, using direct physical impact. One week after injury, 30 μL HBSS (vehicle) or ahMNCs in 30 μL HBSS at a dose of 3 × 10^5^ (low), 1 × 10^6^ (medium), or 3 × 10^6^ (high) were transplanted into the LV of the SCI rats (Figure 2A). To assess the therapeutic effect of these ahMNCs and the relationships between transplantation dose and recovery, locomotor function was evaluated using the open field test once a week for 6 weeks post injury (Figure 2A). Locomotor dysfunction was quantified using the Basso–Beattie–Bresnahan (BBB) score [16]. Although gradual recovery was observed in all groups, recovery of locomotor function was significantly better in the low, medium, and high treatment groups when compared with the vehicle group at all time points (Figure 2B and Table 1). Notably, significant differences in the BBB score were observed between the low and medium groups at 5 and 6 weeks post injury (Figure 2C and Table 1). These results suggest that ahMNCs have significant positive effects on SCI and that the injection dose could influence the treatment outcomes in SCI.

Tissue loss within the injured spinal cords was evaluated using histology at 6 weeks post injury (Figure 2A). The low, medium, and high groups presented with significantly less tissue loss (relative area of cavity) than the vehicle control (Figure 2D,E), which supports the locomotor dysfunction findings as well. In addition, the medium and high groups showed significantly better tissue preservation than the low group (Figure 2E) and there was a significant negative correlation between the BBB score and the overall tissue loss in this model (R = −0.52, Figure 2F). Taken together, these results suggest that the therapeutic effects of ahMNCs may be mediated via the tissue-protective function of ahMNCs.

### 2.3. Alterations in Glial Scar Formation

**Figure 3:**
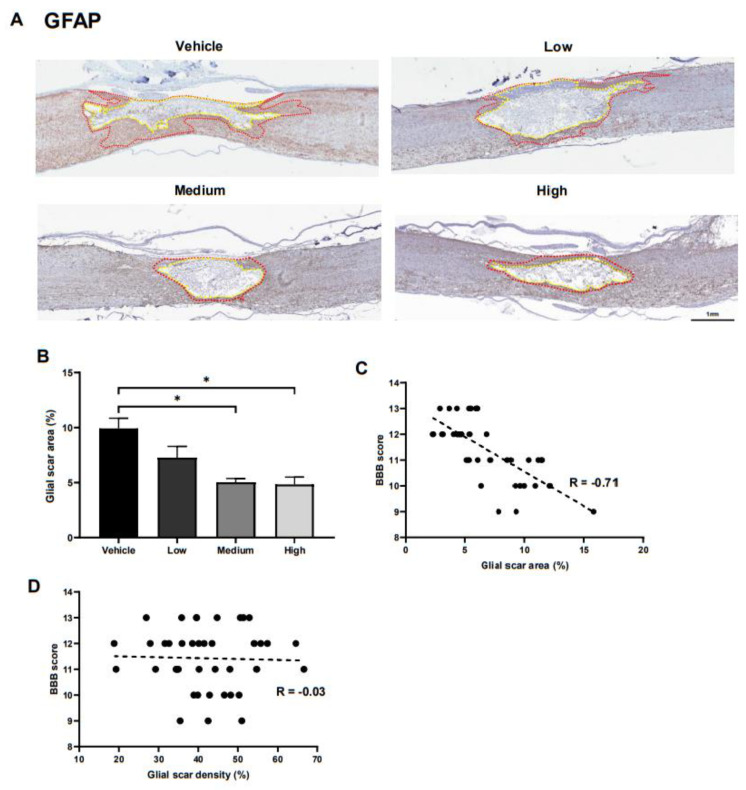
Effects of ahMNC transplantation on glial scar formation. (**A**) Glial scarring was measured using immunohistochemistry against GFAP. Red and yellow lines delineate the outer and inner contours of the glial scar, respectively. Scale bar = 1 mm. (**B**) The relative size of the glial scars was determined and then compared. (**C**) Correlation analysis revealed a significant negative relationship between the glial scar area and the BBB score. (**D**) However, there was no significant relationship between glial scar density and BBB score. Height = Average, Error bar = Standard Error. *, *P* < 0.05.

To elucidate the underlying mechanisms facilitating the ahMNCs tissue-protective functions, we went on to compare the glial scar formation in each of the experimental groups at 6 weeks post injury (Figure 2A). Astrocyte activation and glial scar formation have been shown to be critical during neurological function recovery following SCI [17]. We were then able to accurately identify the glial scar border by separating the astrocytes into three major subcategories based on their GFAP staining (Supplementary Figure S1). Glial scars were shown to include areas that were enriched for GFAP-positive astrocytes, although the individual astrocytes were hard to identify, while there were easily identified individual protoplasmic astrocytes in the gray matter and fibrous astrocytes in the white matter (Supplementary Figure S1). The relative size of the GFAP-positive glial scar (between the yellow and red lines in Figure 3A) was shown to significantly decrease in the medium and high groups when compared to the vehicle group (Figure 3B). In addition, correlation analysis revealed a significant negative relationship between the relative size of the glial scar and the BBB score (Figure 3C). In contrast, the intensity of GFAP immunoreactivity in the glial scar did not influence the BBB score (Figure 3D).

### 2.4. In Vivo Neuroprotective Effects of ahMNCs

**Figure 4:**
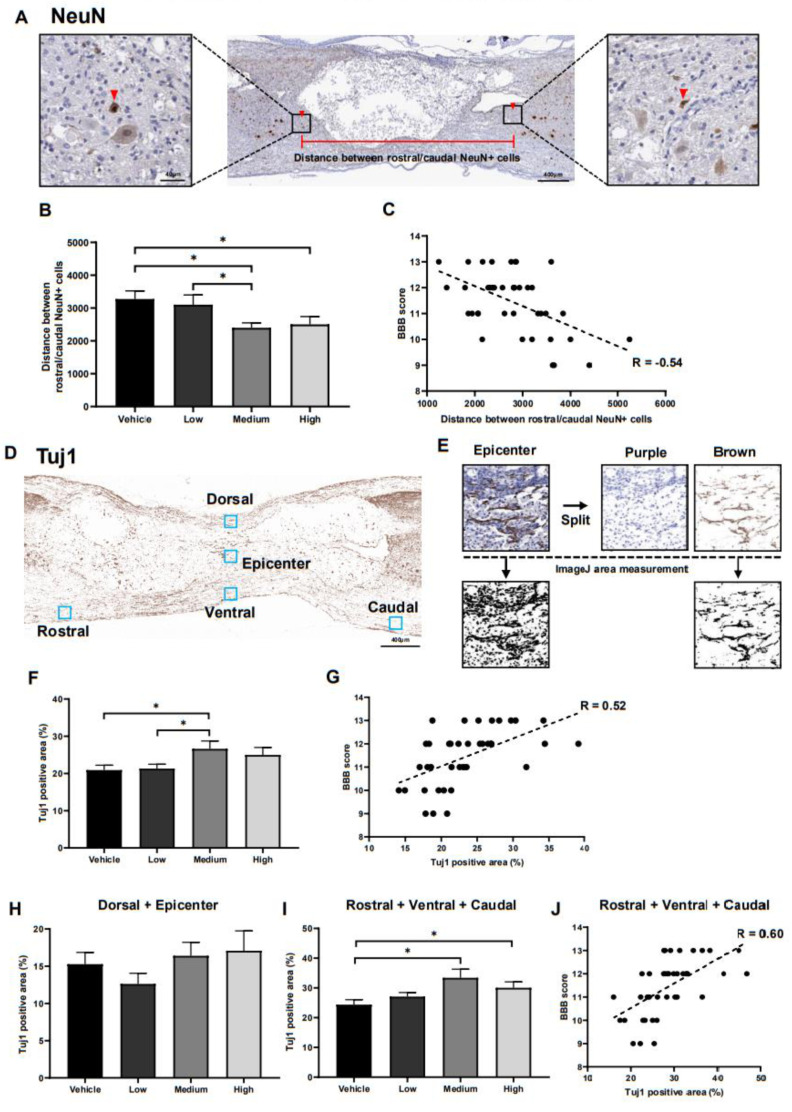
In vivo neuroprotective activities of ahMNCs. (**A**) Immunohistochemistry against NeuN. Red arrows indicate NeuN positive neurons in close proximity to the damaged lesions both rostrally and caudally and the distance between these neurons was evaluated as part of this experiment. Scale bar = 400 µm and 40 µm in the lower and higher magnifications, respectively. (**B**) These distances were quantified and then compared. (**C**) Correlation analysis revealed a significant negative relationship between the distance between the NeuN positive cells and the BBB score. (**D**) Tuj1 immunoreactivity was evaluated in five areas: Dorsal, Epicenter, Rostral, Ventral, and Caudal. (E) Imaging revealed which of these areas were experiencing this immunoreactivity. (**F**) The relative intensity of these images was compared to determine the extent of Tuj1 immunoreactivity in each area. (**G**) Correlation analysis revealed a significant positive relationship between the relative area of Tuj1 reactivity and the BBB score. (**H**,**I**) These immunoreactive areas were independently quantified for the Dorsal and Epicenter lesions (**H**) and Rostral, Ventral, and Caudal lesions (**I**) and then compared. (**J**) Correlation analysis revealed a significant positive relationship between the Tuj1-positive area in the Rostral, Ventral, and Caudal lesions and the BBB score. Height = Average, Error bar = Standard Error. *, *P* < 0.05.

The in vivo neuroprotective effects of ahMNC transplantation were evaluated using immunohistochemistry against NeuN and Tuj1 on tissues collected 6 weeks post injury (Figure 2A). First, the nearest distance between the rostral and caudal NeuN-positive neuronal nuclei was measured since neurons in the damaged tissue degenerate (Figure 4A). This distance was significantly reduced in the medium treatment group when compared to the vehicle control and low group, while the high group also showed a significant reduction in this distance when compared to the vehicle control (Figure 4B). In addition, our analysis revealed that there is a significant negative correlation between this distance and the BBB score (R = −0.54, Figure 4C).

We evaluated Tuj1 expression to determine the effects of ahMNC transplant on both the axons and dendrites in injured rats (Figure 4D,E). Tuj1 expression was much higher in the medium treatment group when compared with both the vehicle control and the low group (Figure 4F) and there was a significant positive correlation between Tuj1 immunoreactivity and the BBB score (R = 0.52, Figure 4G). Despite this, there were no significant differences in Tuj1 staining between the high dose treatment group and any of the other groups (Figure 4F). Tuj1 expression was analyzed at five points near the injury site: dorsal, epicenter, rostral, ventral, and caudal (Figure 4D,E). The dorsal and epicenter points were used to evaluate directly damaged sites where axons and dendrites were disrupted during the SCI. This means that the presence of Tuj1 positive fibers at these points may indicate regrowth of the fibers in these sections while the rostral, ventral, and caudal points were evaluated as indirect measures of injury. Tuj1-positive fibers at these sites would most likely be the result of fiber survival and not regeneration. Unfortunately, there were no significant differences in Tuj1 reactivity in the dorsal and epicenter tissues between any of the groups (Figure 4H), but both the medium and high treatment groups presented with significantly increased Tuj1 expression in the rostral, ventral, and caudal positions (Figure 4I). In addition, there was a significant degree of correlation between Tuj1 reactivity in the rostral, ventral, and caudal points and the BBB score (R = 0.60, Figure 4J).

Taken together these results support the hypothesis that ahMNCs exert their SCI therapeutic effect via their protection of the neurons and fibers near the injury site.

### 2.5. Alteration in Oligodendrocytes

**Figure 5:**
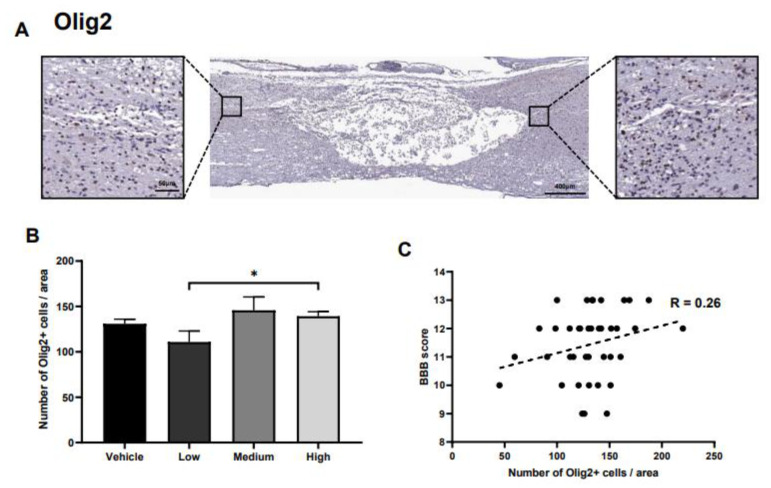
Effects of ahMNCs transplantation on oligodendrocytes. (**A**) Immunohistochemistry against Olig2. Scale bar = 400 and 50 µm in the lower and higher magnifications, respectively. (**B**) The number of Olig2-positive oligodendrocytes was quantified and compared. Scale bar = 400 and 50 µm in the lower and higher magnifications, respectively. (**C**) Correlation analysis between the number of Olig2 positive oligodendrocytes and the BBB score revealed the relationship between these two parameters. Height = Average, Error bar = Standard Error. *, *P* < 0.05.

Olig2-positive oligodendrocytes were identified and quantified at the rostral and caudal points (Figure 5A). Although the number of oligodendrocytes increased significantly when comparing the high- and low-dose treatment groups, the number of these cells in the vehicle control was not significantly different from any of the other groups (Figure 5B). In addition, the correlation between Olig2-positive oligodendrocytes and the BBB score was relatively low (Figure 5C).

### 2.6. Effects of ahMNCs on Angiogenesis

**Figure 6:**
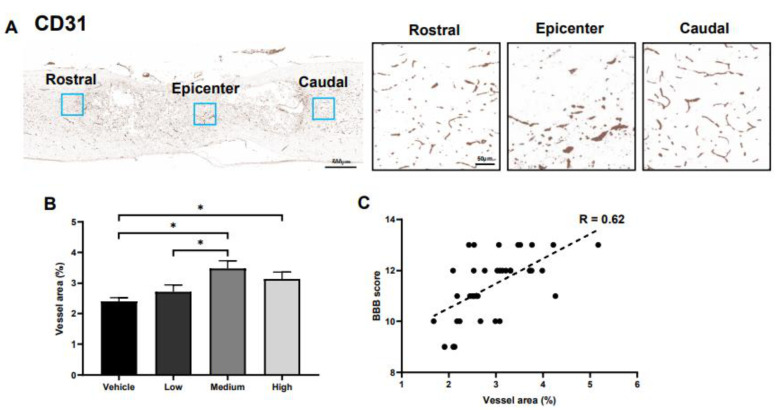
Effects of ahMNCs transplantation on angiogenesis. (**A**) Immunohistochemistry against CD31. Scale bar = 500 and 50 µm in the lower and higher magnifications, respectively. (**B**) The areas with CD31-positive vessels were quantified and compared. Scale bar = 400 and 50 µm in the lower and higher magnifications, respectively. (**C**) Correlation between the relative area and BBB score revealed a significant positive relationship between these parameters. Height = Average, Error bar = Standard Error. *, *P* < 0.05.

We previously reported that transplantation of ahMNCs significantly increased vessel density in damaged spinal cords [11]. To evaluate the angiogenic effects of ahMNCs, the vessel area was measured using immunohistochemistry against CD31, an endothelial cell-specific marker, at three points: rostral, epicenter, and caudal regions (Figure 6A). The vessel area of the medium group was significantly broader than that of the vehicle and low group (Figure 6B) and the high group also demonstrated a significant increase in the vessel area when compared to the vehicle control (Figure 6B). In addition, there was a significant correlation between the vessel area and the BBB score (R = 0.62, Figure 6C).

### 2.7. In Vivo Distribution of Transplanted ahMNCs

**Figure 7:**
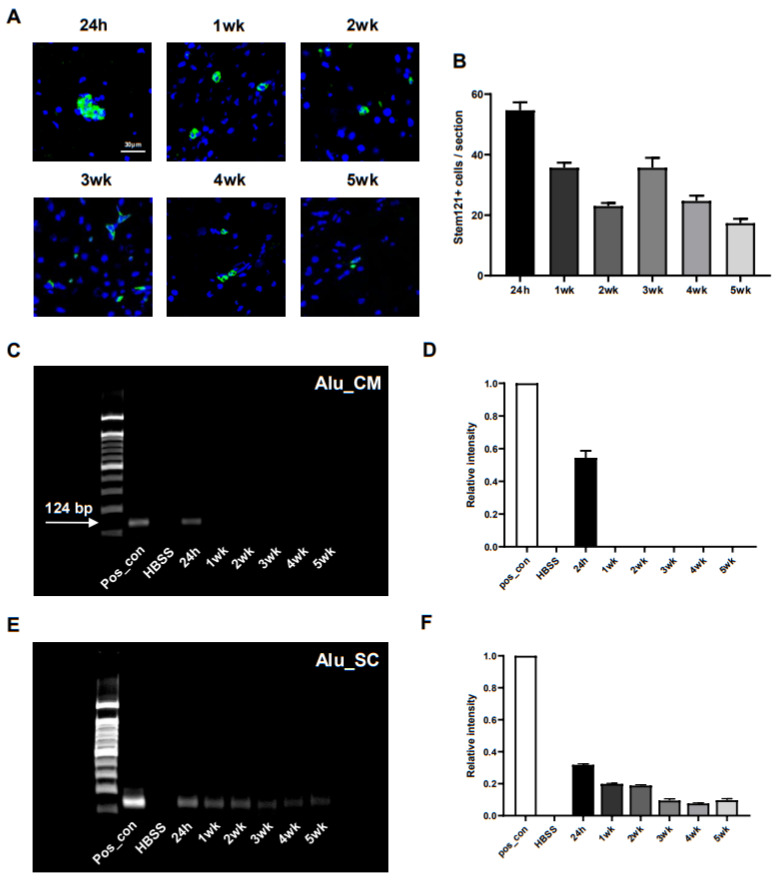
In vivo distribution of transplanted ahMNCs. (**A**) Immunohistochemistry allowed for the detection of ahMNCs which present with human cytoplasm (green). Blue = Nuclei. Scale bar = 30 µm. (**B**) The average number of human cells in three serially derived sections were quantified in each animal and then compared. (**C**) Human-specific Alu sequences (124 base pairs) were amplified from several brain tissues including the CM using PCR. (**D**) Relative intensities of the PCR products were quantified. (**C**) Human-specific Alu sequence was amplified by PCR in several tissues including the SCI lesion. (**D**) Relative intensities of the PCR products were quantified. Height = Average, Error bar = Standard Error.

The evaluation of the survival and distribution of transplanted cells is critical when determining the safety and efficacy of any novel therapeutic process [18]. For this reason, we designed an experiment that allowed us to trace our injected ahMNCs for 24 h, 1 week, 2 weeks, 3 weeks, 4 weeks, and 5 weeks after transplantation using a human cytoplasm-specific antibody (Stem121) and PCR of human specific-Alu (*n* = 3 for each group). Immunohistochemistry (Figure 7A) revealed that human cytoplasm-positive cells were observed in the injured spinal cord of all animals until 5 weeks after injection, although the number of cells decreased with time (Figure 7B) while human-specific Alu sequences could only be detected in most areas of the brain, including the cisterna magna, for the first 24 h post-transplant (Figure 7C,D). In contrast, these sequences were easily identified in the injured spinal cord for up to 5 weeks after the initial injection, although the relative amount of PCR product decreased continuously (Figure 7E,F). These results indicate that ahMNCs transplanted into the LV may migrate through the cisterna magna to the injured spinal cord and then persist in this niche for up to 5 weeks.

## 3. Discussion

The results of this study highlight five important observations. First, ahMNCs isolated from patients with hemorrhagic stroke were successfully applied in a preclinical model of SCI. Previously, ahMNCs were cultured from temporal lobe epilepsy tissues, specifically, focal cortical dysplasia (FCD) type IIIa [9,10,11]. In FCD type IIIa, mild localized structural distortion of the cerebral cortex within the temporal lobe is accompanied by hippocampal sclerosis [19,20]. Although most of the cerebral cortex in surgical samples from FCD type IIIa is structurally normal, neural cells in the temporal lobe have experienced multiple abnormal electrical events. Despite this, our previous studies [10,11] did not report any seizure-like behaviors in the animals that underwent transplantation with these cells. However, preclinical experiments might not evaluate and exclude all potential unwanted side effects. In contrast, ahMNCs from hemorrhagic stroke were not exposed to seizure. Since ahMNCs from hemorrhagic stroke share key features with ahMNCs from temporal lobe epilepsy, including the expression of NSC markers and differentiation potential into multiple kinds of neural cells, ahMNCs from hemorrhagic stroke might be a promising alternative source for these NSCs in clinical applications.

Second, ahMNCs transplanted into the LV of SCI animals successfully migrated to the lesion in the thoracic spinal cord and survived in this niche for at least 5 weeks post transplantation. Direct injection of stem cells into the injured spinal cord is common in preclinical evaluations [21,22], however, this is not easily translated in the clinical setting as it might provoke secondary injuries to the damaged and/or nearby spinal cord during operation [12]. Moreover, any physical damage to the inflammatory tissue could potentiate the immune reaction, which in turn may exacerbate the existing SCI. Given this, we previously compared indirect injection routes, including transplantation into the LV and CM, as an alternative to this direct injection and determined that both routes allow for the stem cells to be delivered to the SCI lesion [23]. We identified that cellular distribution via cerebrospinal fluid (CSF) within injured spinal cord tissues was as efficient as that of the undamaged spinal cord when cells were injected into the LV [24]. Our results from this study further suggest that ahMNCs injected intracerebroventricularly can migrate to the SCI lesion and survive for up to 5 weeks post transplantation. This method is clinically applicable making this a necessary first step in supporting these approaches in clinical settings.

Third, the in vivo migration of ahMNCs into the damaged spinal cord was specific. In mammalian CNS, both human and non-human NSCs have the innate ability to proliferate and move to injured and/or degenerating brain areas [25]. This is facilitated by the various factors produced during inflammation which signals the NSCs to home to and aggregate at sites of CNS injury [26]. Researchers have previously reported extensive migration of NSCs following nonspecific targeted intramedullary injections in both preclinical and clinical studies [27,28]. Furthermore, neural progenitor cells (NPCs) transplanted using an intra-arterial route clearly localized to the ischemic hemisphere of the brain following stroke [29]. When ahMNCs were transplanted into the LV of the SCI animals, the CM acted as a gateway allowing the ahMNCs to travel to the damaged spinal cord. At 24 h after transplantation, human-specific Alu sequences were detected in both the brain lesion, including the CM, and the spinal cord. However, 1 week after transplant, PCR could not amplify these sequences in the brain tissues. Given the fact that PCR amplification is one of the most sensitive detection methods available our results strongly suggest that ahMNCs transplanted into the LV did not settle in the normal brain tissue near the CM. The specific migration of these cells to the damaged spinal cord supports the use of this indirect transplantation route in future preclinical and clinical studies.

Fourth, primary cultured ahMNCs exhibit significant preclinical therapeutic effects for SCI. The histological analysis revealed four important alterations to these lesions linked to the presence of the ahMNCs. (1) Gliosis: Glial scars have long been considered the major barrier to axonal regeneration [30,31]. However, recent studies also reported that proper, not excessive, glial scarring leads to reduced axonal dieback and promotes axon regeneration after SCI [32,33]. In this study, we identified a strong negative correlation between glial scar size and functional recovery. This suggests that ahMNCs might exert their therapeutic effects by preventing excessive gliosis. (2) Neuroprotection: Immunohistochemistry against NeuN and Tuj1 showed that the groups injected with ahMNCs had more live neurons and nerve fibers near the SCI lesions. The neuroprotective functions of ahMNCs are mediated by their paracrine effects, although the exact molecules used to exert these neuroprotective effects remain unknown. (3) Angiogenesis: In this study, ahMNCs promoted angiogenesis in the SCI lesion, and this was also observed in a previous study [11]. New vessel formation might promote elimination of myelin debris [34], which could inhibit axon regeneration [35,36] and remyelination [37,38].

Fifth, the transplanted dose of ahMNCs influences their therapeutic efficacy in SCI. Here, we chose to evaluate three injection doses: low (3 × 10^5^), medium (1 × 10^6^), and high (3 × 10^6^) dose and observed a maximum therapeutic effect in the medium dose. Since cell suspensions at high concentrations (3 × 10^6^ cells in 30 μL HBSS) are relatively sticky, we hypothesize that the ahMNCs in this injection line might not be transplanted properly. A recent study suggested that a higher dose of fetal NSCs increases successful engraftment, neuronal survival, and axonal regeneration in animal models of SCI leading to better functional recovery [39]. However, the higher dose of fetal NSCs used in the study was 1.5 ×10^6^, which is more like the medium dose used in this study. In contrast, a meta-analysis of the available data suggested that a higher dose of autologous olfactory ensheathing cells induces more neurotoxic effects [40]. Transplantation dose could also affect the in vivo differentiation of NSCs [41,42]. Therefore, preclinical doses may not translate to clinical settings and these concentrations should be evaluated considering several factors. Our results suggest that although the medium dose of ahMNCs showed the best therapeutic effects for SCI in our hands, this should be experimentally validated in other models prior to clinical translation.

Taken together, the results of this study show that ahMNCs derived from hemorrhagic stroke have significant therapeutic potential in a preclinical model of SCI and that these effects might be mediated by the modulation of gliosis, neuroprotection, and/or angiogenesis in the damaged spinal cord. Considering that ahMNCs indirectly injected into the LV migrated specifically into the SCI lesion and that the transplantation dose influenced their therapeutic effect, we can say that the results of this study may help with the clinical evaluation of these technologies in the future.

## 4. Materials and Methods

### 4.1. Study Approval and Animal Care

Informed written consent was obtained from patients with hemorrhagic stroke according to the guidelines approved by the Institutional Review Board of the Samsung Medical Center (SMC, Seoul, South Korea) (IRB File No. 2016-11-085-003) allowing us to collect surgical samples from these participants. All animal studies were approved by the Institutional Animal Care and Use Committee (IACUC) from the Laboratory Animal Research Center (LARC) at the Sungkyunkwan University School of Medicine (SKKUIACUC2018-05-06-3). Animal experiments were conducted in accordance with the Guide for the Care and Use of Laboratory Animals from the Institute for Laboratory Animal Research [43].

### 4.2. Cell Culture

Brain tissue samples obtained from patients with hemorrhagic stroke were used in this study, and the primary culture of these specimens was completed as previously described [9]. Briefly, within 2 h of surgery, surgical specimens were weighed and minced and then partially digested using an enzyme solution containing 10 units/mL papain (Sigma, St. Louis, MO, USA), 0.1 mg/mL DNase I (Roche, Basel, Switzerland), and 4 mg/mL d, l-cysteine (Sigma) at 37 °C for 20 min. After mild trituration, cells were placed in phosphate-buffered saline (PBS) and filtered through a cell strainer (40 μm, Corning, NY, USA), and then used for adherent culture in DMEM/F12 (Gibco, Grand Island, NY, USA) supplemented with 1% B27 supplement (Xenofree, Gibco), 5 μg/mL gentamicin (Gibco), 1% N-2 supplement (Gibco), 50 ng/mL epidermal growth factor (EGF) (R&D Systems, Minneapolis, MN, USA), 50 ng/mL basic fibroblast growth factor (bFGF) (R&D Systems), and 0.5% fetal bovine serum (FBS, Gibco). For differentiation, cells were cultured in DMEM/F12 supplemented with 1% B27 supplement, 1% N-2 supplement, 5 μg/mL gentamicin, 0.5% FBS, and 0.5 mM 3-isobutyl-1-methylxanthine (IBMX) (Sigma) for 3 days.

### 4.3. Immunocytochemistry

Cells were fixed in ice-cold 4% paraformaldehyde (PFA, Biosesang, Gyeonggi, South Korea) at room temperature (RT) for 20 min, permeabilized in 0.2% Triton X-100 for 20 min, blocked using blocking buffer containing 2% normal goat serum (NGS, Abcam) and 1% bovine serum albumin (BSA, GenDEPOT, Barker, TX, USA) in PBS, and then incubated with the following primary antibodies: Nestin (1:200, Novus Biologicals, Littleton, CO, USA); Ki67 (1:250, Abcam, Cambridge, MA, USA); Tuj1 (1:1000, Abcam); GFAP (1:2000, Abcam), Claudin11 (1:250, Abcam) at 4 °C overnight. Samples were rinsed and then treated with Alexa Fluor 594-conjugated goat anti-Rabbit IgG antibodies (1:500, Abcam) and the nuclei were counterstained with DAPI for 10 min at RT. After mounting, the cells were examined using confocal laser scanning microscopy (BIORP, Leica, Wetzlar, Germany).

### 4.4. Spinal Cord Injury Animal Model

Adult female Sprague-Dawley rats (10-week-old, 250–300 g, Orient Bio., Sungnam, South Korea) were anesthetized for 3 min using 5% isoflurane (Hana Pharm, Seoul, South Korea) and this anesthesia was maintained at 2.5–3% isoflurane throughout the surgical procedure. The thoracic spinal cords in the dura mater (T9–T10) were exposed by laminectomy and a 200 kilodyne force was delivered to the T9 spinal cord using an Infinite Horizon (IH) Impactor (Precision Systems & Instrumentation, Lexington, KY, USA). After contusion, the muscle, subcutaneous layer, and skin were sutured, and the animals were treated with 10 mg/kg Ketoprofen (Uni Biotech, Seoul, South Korea) via a single intramuscular injection a day for 2 days to reduce pain after the surgery. Bladder compression was performed twice a day until spontaneous micturition was achieved. Motor function of the hind limbs was evaluated using the Basso–Beattie–Bresnahan (BBB) locomotor rating test on an open field [16] once a week. 

### 4.5. Cell Transplantation

Animals with BBB scores of between 4 and 6 at day 7 post-injury were randomly divided into four groups: vehicle, low, medium, and high groups (*n* = 10 for each group). Then these animals were subjected to LV transplantation as previously described [24]. Briefly, under isoflurane anesthesia, rats were fixed in a stereotaxic device (Model 900 small animal stereotaxic instrument, KOPF stereotaxic, Tujunga, CA, USA). The transplantation site was anterior/posterior (AP): 1.4 mm, dorsal/ventral (AP): −0.8 mm, dorsal/ventral (DV): 3.8 mm from the bregma. Then 30 µL of HBSS (Gibco) (for the vehicle group), 3 × 10^5^ ahMNCs in 30 μL HBSS (for the low group), 1 × 10^6^ ahMNCs in 30 μL HBSS (for the medium group), or 3 × 10^6^ ahMNCs in 30 μL HBSS (for the high group) were injected into the LV over a 10 min period using a Hamilton syringe (26G, Hamilton Company, Reno, NV, USA), and a syringe pump (LEGATO™111, KD Scientific, Holliston, MA, USA). ahMNCs were all grown to in vitro passage 6 (P6) and the needle was left in the injection sited for 5 min after the end of the transplant to prevent leakage, and then removed at a speed of 1 mm/min. Ketoprofen (10 mg/kg) was administered via intramuscular injection to reduce pain after surgery, and immunosuppression was induced using cyclosporine A (CSA, 10 mg/kg) (Chong Kun Dang Pharmaceutical Corp., Seoul, South Korea), which was administered subcutaneously every day from 24 h prior to transplant to 4 weeks after transplant. The CSA concentration was reduced by half every week until the animals were fully weaned.

To evaluate the distribution of transplanted ahMNCs, 1 × 10^6^ ahMNCs (P6) in 30 μL of HBSS were injected into the LV of 18 SCI animals at 7 days after injury using the same experimental procedures described above.

### 4.6. Tissue Processing

The rats were sacrificed in a CO_2_ chamber and the spinal cord injury site was identified. The tissues were then embedded in paraffin as described previously [44] and the distribution of the transplanted ahMNCs was evaluated. The rostral segments of the spinal cord injury sites were fixed in PFA for 12 h, incubated in a 30% sucrose solution at 4 °C for 24 h, and then submerged in OCT compound (Leica), and frozen on dry ice. In addition, various brain regions including the cisterna magna were harvested for PCR. Caudal segments from the spinal cord injury sites and the various brain tissues, including the cisterna magna, were cryopreserved at −80 °C until genomic DNA extraction for PCR.

### 4.7. Immunohistochemistry

Paraffin blocks were sectioned (4 μm thick) and then placed on silane-coated microscope slides (Muto Pure Chemicals Co., Ltd., Tokyo, Japan). These slides were then heated on a slide warmer (Lab-line Instruments USA, Dubuque, IA, USA) at 65 °C for 30 min, then deparaffinized, rehydrated, and then boiled in the target retrieval solution (Dako, Carpentaria, CA, USA) four times for 5 min each to allow for antigen retrieval. The slides were incubated in 0.3% ammonia in 70% methanol for 1 h and then washed in 50% methanol for 10 min and incubated in 3% hydrogen peroxide in methanol for 12 min to quench any endogenous peroxidase activity. Blocking was performed by incubating these slides in 5% BSA (Abcam) and 2% NGS (GenDEPOT) in a peroxidase-blocking solution (Dako) for 1 h at RT. Primary antibodies were then added and incubated at 4 °C overnight: GFAP (1:2000, Abcam), NeuN (1:500, Millipore, Temecula, CA, USA), Tuj1 (1:1000, Abcam), CD31(1:2000, Abcam), and Olig2 (1:250, Millipore). These slides were then incubated with HRP-conjugated secondary antibody (Abcam) for 1 h at RT and then reacted with 3,3′-diaminobenzidine tetrahydrochloride (DAB, Dako) at RT for 30 s (NeuN), 40 s (GFAP and Tuj1), or 60 s (CD31 and Olig2).

Frozen blocks were cryo-sectioned (8 μm thick) and permeabilized with 0.2% Triton X-100 for 20 min at RT and placed on slides. These slides were incubated in 3% hydrogen peroxide in methanol for 12 min at RT and blocked with 2% BSA and NGS in PBS for 1 h at RT. Next, anti-human cytoplasm antibody (Stem121, 1:500, Takara Bio, Shiga, Japan) and anti-Nestin, Tuj1, GFAP, or Olig2 antibody were added and incubated overnight at 4 °C. These sections were then incubated with Alexa Fluor 488-conjugated goat anti-mouse IgG antibody (1: 500, Invitrogen, Carlsbad, CA, USA) for 2 h at RT and then the sections were covered with coverslips and mounting medium containing DAPI (Vector Laboratories, Burlingame, CA, USA).

### 4.8. Image Analysis

Spinal cord sections were visualized using DAB and scanned. The colors in the scanned images were separated into purple and brown using QuPath (Queen’s University Belfast, Northern Ireland, UK) image analysis software (Figure 4E) [45]. Computational color deconvolution was applied to separate the hematoxylin and 3,3′-diaminobenzidine (DAB) staining and the brown immunohistochemical staining was analyzed using ImageJ software (NIH Image, Bethesda, Maryland, USA).

### 4.9. Polymerase Chain Reaction

Genomic DNA was extracted using the DNeasy^®^ Blood and Tissue Kit (250) (Qiagen, Hilden, Germany) according to the manufacturer’s instructions. Alu PCR was completed using the following primers: forward = 5′-TCAGGAGATCGAG-ACCATCCC-3′′and reverse = 5′-TCCTGCCTCAGCCTCCCAAG-3′ [46]. PCR was performed using an Ex Taq DNA polymerase kit (Takara-Bio, Tokyo, Japan) and the reaction mixture comprised 5 μL of 10× Ex Taq Buffer, 4 μL of dNTP mixture, 1 μL (10 pM) of each primer, 2 μL (50ng) of gDNA template, 0.25 μL of TaKaRa Ex Taq and 36.75 μL of sterile distilled water. Cycling conditions were as follows: 50 °C for 20 s, 95 °C for 15 min, 40 cycles of denaturation at 95 °C for 15 s, annealing at 62 °C for 30 s, and extension at 72 °C for 30 s. The PCR product (20 µL) was electrophoresed on a 2% agarose gel with a 100-bp marker and the normalized intensity from these gels was quantified using ImageJ software. The positive control, genomic DNA, was extracted from the ahMNCs prior to experimentation.

### 4.10. Statistical Analysis

All data are presented as the mean ± standard error (SEM). Data were analyzed using the two-tailed Student’s t-test and P-values of less than 0.05 were considered significant. 

## Figures and Tables

**Table 1:** BBB scores for each of the experimental groups.

	1 wk		2 wk		3 wk		4 wk		5 wk		6 wk	
	Average	SEM	Average	SEM	Average	SEM	Average	SEM	Average	SEM	Average	SEM
Vehicle (*n* = 10)	4.5	0.22	8.8	0.20	9.8	0.13	10.1	0.18	10	0.26	10	0.26
Low (*n* = 10)	4.7	0.21	9.8	0.36	10.7	0.30	11.1	0.38	11.3	0.33	11.4	0.27
Medium (*n* =10)	4.5	0.22	10.6	0.16	11	0.37	11.8	0.25	12.2	0.20	12.3	0.21
High (*n* = 10)	4.9	0.10	10.2	0.25	11.3	0.30	11.5	0.37	11.8	0.29	12	0.30

## Data Availability

The data presented in this study are available on request from the corresponding author.

## Supplementary Materials

The following are available online at https://www.mdpi.com/1422-0067/22/5/2579/s1, Figure S1: Determination of glial scarring.
