# Supplementary figures and images for: Optimal Preclinical Conditions for Using Adult Human Multipotent Neural Cells in the Treatment of Spinal Cord Injury
Source: Int J Mol Sci. 2021 Mar 4;22(5):2579. doi: 10.3390/ijms22052579 (PMC7961778; doi:10.3390/ijms22052579)

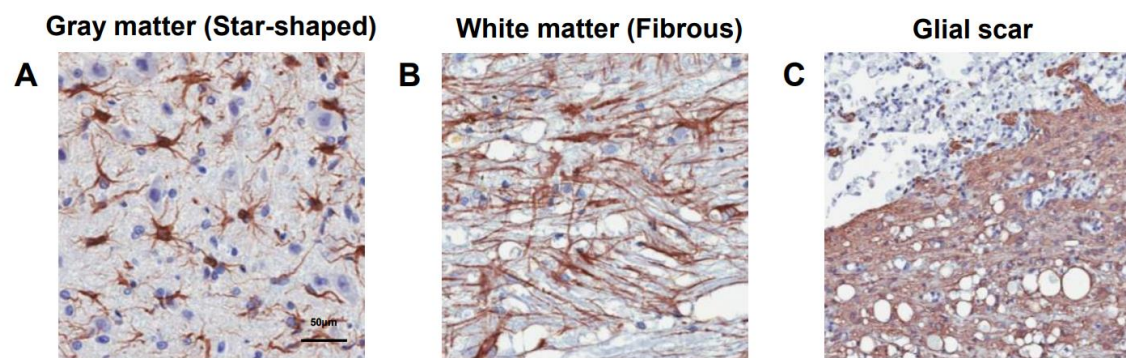

**Figure S1.** Determination of glial scarring.

Supplement: Supplementary file 1 [file ijms-22-02579-s001.pdf]
